# Human parvovirus B19-induced anaemia in pre-school children in Ilorin, Nigeria

**DOI:** 10.4102/ajlm.v7i1.615

**Published:** 2018-05-10

**Authors:** Oluwaseyi S. Ashaka, Olajide O. Agbede, Adesuyi A. Omoare, Samuel K. Ernest

**Affiliations:** 1 Department of Medical Microbiology and Parasitology, College of Health Sciences, University of Ilorin, Ilorin, Kwara State, Nigeria; 2 Department of Paediatrics and Child Health, College of Health Sciences, University of Ilorin, Ilorin, Kwara State, Nigeria

## Abstract

Sera collected from 57 anaemic and 115 non-anaemic age-matched pre-school children in Ilorin, Nigeria, between November 2014 and December 2015 were assayed for human parvovirus B19-specific IgM antibodies by using the enzyme linked immunosorbent assay technique. A total of 17 (29.8%) anaemic children and 18 (15.7%) non-anaemic children were positive for parvovirus B19 infection. Infection with parvovirus B19 is common in this population, and screening for the virus during differential diagnosis is recommended.

## Introduction

Parvovirus B19 is a small, non-enveloped, single-stranded DNA virus.^1^ The nucleocapsid of the virus has icosahedral symmetry. Parvovirus B19 is transmitted through the respiratory routes and through contact with infected blood and blood products to susceptible individuals, including children with underdeveloped immune systems. Risk factors associated with parvovirus B19 infection include blood transfusion, circumcision, tribal marks and scarification.^2^

Interest in parvovirus B19 has increased because of the burden of anaemia among children in developing countries where multiple other causes of anaemia abound.^3,4^ The cause of anaemia among pre-school children living in sub-Saharan Africa has been associated with malaria, haemoglobinopathy, iron deficiency, folic acid deficiency, vitamin B12 deficiency, vitamin A deficiency, HIV infection, helminthic infestation, sickle cell disease and autoimmune haemolytic anaemia^3^. In Nigeria, anaemia may also be due to iron deficiency resulting from insufficient dietary intake in children. The majority of hospital admissions for children under the age of five years have been attributed to malaria.^5^ In addition, sickle cell disease contributes significantly to the burden of anaemia in this population.^6^ Some reports on the co-infection of *Plasmodium sp*. with parvovirus B19 exists and it is known that both agents affect red blood cells.^7^

The association of human parvovirus B19 with anaemia, especially among pre-school children, has been established by other studies, which have reported levels between 7% and 14%.^4^ Parvovirus B19 preferentially targets young erythrocytes and temporarily suppresses red blood cell production.^8^ Parvovirus infection in anaemic pre-school children contributes to anaemia and could also be responsible for increased childhood morbidity and mortality among children with other underlying causes of anaemia.^9^ This raises the index of suspicion that parvovirus B19 may be involved in increasing the burden of anaemia in pre-school children.

This study was initiated at the University of Ilorin Teaching Hospital and the Children’s Specialist Hospital to determine the status of pre-school children with anaemia regarding possible infection with human parvovirus B19.

## Methods

### Ethical considerations

This study was conducted in compliance with the Helsinki Declaration and was approved by the Health Research and Ethics Committee of the University of Ilorin Teaching Hospital and the Hospital Management Bureau at the Children’s Specialist Hospital (approval number ERC PAN/2014/09/1314). The parents or guardians of participants gave written informed consent before their children were enrolled in the study. All data were analysed anonymously throughout the study. A semi-structured questionnaire was administered to obtain relevant information on the children’s sociodemographic characteristics; evidence of rash and laboratory findings were documented.

### Study design and participants

This was an observational, prospective, cross-sectional, hospital-based case control study. Patients were consecutively recruited from among pre-school children who visited the University of Ilorin Teaching Hospital or the Children’s Specialist Hospital between November 2014 and December 2015. All consecutive patients visiting either hospital were recruited. All patients recruited resided in Ilorin, a city characterised by communal living, an open sewage system, and challenges for water utilisation and sanitation in its underdeveloped urban areas.

All patients presenting with anaemia at either hospital were identified using a protocol set up by the paediatric clinics that classified patients with haematocrit values of ≤ 30% as anaemic (cases) and patients with haematocrit values of > 30% as non-anaemic (controls).^10^ Controls with a significant fever (temperature ≥ 38 °C) were excluded from the study. It should be noted that the type of anaemia examined in this study was based on reticulocyte count with normal range value of 0.8% – 2.2% (Haematocrit value: 31.7% – 39.6%). Patients with sickle cell disease were deliberately excluded from this study.

### Serologic testing

Serum samples were collected from all participants. Three millilitres of blood was drawn from each participant by a paediatrician. Aliquots were decanted into tubes without anticoagulant for parvovirus B19 immunoglobulin M (IgM) serology and into EDTA anticoagulant tubes for haematocrit analysis to confirm anaemia. Blood sample bottles were labelled with a unique sample code starting with ‘A’ for cases (children with anaemia) and a code starting with ‘C’ for controls.

Determination of haematocrit was done by centrifugation using standard procedures at a fixed speed of 11 000 revolutions per minute (rpm) for 10 minutes (Hawksley & Sons Ltd, Sussex, United Kingdom). Serum samples were separated by centrifugation at 400 revolutions per minute and stored at -20°C for three months after which the enzyme linked immunosorbent assay (ELISA) was performed. The ELISAs used a recombinant VP1 protein to capture IgM antibody response to viral capsid protein-1 antigen of human parvovirus B19 (Vircell, Granada, Spain). All assays were performed according to the manufacturer’s written instructions (Vircell, Granada, Spain). Parvovirus antigen that contained inactivated parvovirus B19 antigen (VP2 obtained in baculovirus) was used. Positive control which contained serum with parvovirus antigen and negative control contained serum without parvovirus antigen were tested with each batch of patient samples.

### Statistical analysis

The generated data were systematically analysed as appropriate for mean, proportion and chi-square test using SPSS for Windows statistical software version 18.0 (SPSS Inc., Chicago, Illinois, United States; 2009). The chi-square was used to determine differences between groups and a one-sided probability of < 0.05 was considered statistically significant. Every child meeting the case definition was enrolled in the study. Age-matched children (±5 months from the age of a case) were selected as controls from among children meeting the inclusion criteria who attended the health facility during the study period.

## Results

A total of 172 pre-school children younger than age five participated in the study; 92 were boys, 80 were girls and the mean age for all children was 1.66 years. A total of 21 (22.82%) boys and 14 (17.5%) girls were positive for human parvovirus B19 infection. A total of 57 participants were anaemic, of whom 17 (29.8%) were positive for parvovirus B19 infection; 115 participants were non-anaemic, of whom 18 (15.7%) were positive for parvovirus B19 infection (Table 1). A large percentage of anaemic participants (*n* = 40; 70.2%) were negative for parvovirus B19 infection. Overall, 97 (84.4%) participants had no anaemia and no parvovirus B19 infection. Only two of the 17 anaemic children positive for parvovirus B19 infection had visible rash.

**TABLE 1:** Comparison of human parvovirus B19 infection among anaemic and non-anaemic children, Ilorin, Nigeria, November 2014–December 2015 (*N* = 172)

Health status†	Parvovirus positive‡ IgM	Parvovirus negative‡ IgM
Anaemic	17	40
Non-anaemic	18	97

X^2^ = 4.72, *p* value = 0.03

† , Anaemic = haematocrit value of ≤ 30%; non-anaemic = haematocrit value of > 30%.

‡ , As determined by an enzyme linked immunosorbent assay technique that used a recombinant VP1 protein to capture IgM antibody response to viral capsid protein-1 antigen of human parvovirus B19.

**FIGURE 1:**
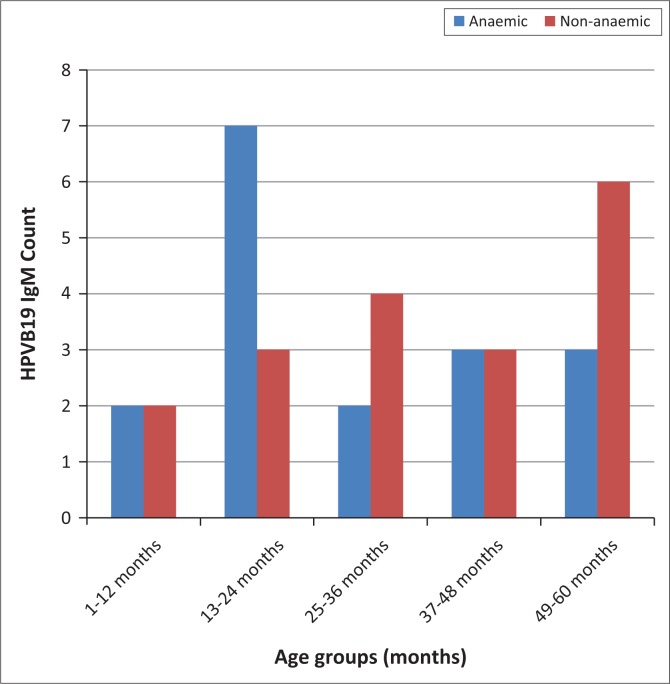
Distribution of human parvovirus B19 infection among age groups of anaemic and non-anaemic pre-school children in Ilorin, Nigeria, November 2014–December 2015 (*N* = 172).

Prevalence of parvovirus B19 infection among anaemic participants was highest among the 13–24-month age group (*n* = 7) (Figure 1). Prevalence of parvovirus B19 infection among non-anaemic participants was highest among the 49–60 month age group (*n = 6*).

## Discussion

Our study found that both anaemic and non-anaemic pre-school children living in Ilorin, Nigeria tested positive for human parvovirus B19 infection. Of the 57 anaemic children, 17 (29.8%) were parvovirus B19-positive, and of the 115 non-anaemic children, 18 (15.7%) were parvovirus B19-positive.

Few studies have reported on prevalence of parvovirus B19 infection in children. The prevalence of parvovirus B19 infection in such studies ranges between 5% and 14.3% among children with sickle cell anaemia.^11,12^ This is lower than the prevalence of 29.8% we found among pre-school children with anaemia in Ilorin. Our study also found that parvovirus B19-infection prevalence was higher in children with anaemia when compared to non-anaemic controls and that the difference was statistically significant. This difference may be as a result of recent infection with human parvovirus B19 in this study population. A study in Italy reported that parvovirus B19 can persist in immunocompetent symptomatic and non-symptomatic individuals by the presence of viral DNA in different tissue but this was observed in the absence of viraemia and anti-B19 IgM.^13^ These results have implications for children under the age of five years, because such children are vulnerable to the detrimental effects of anaemia. The presence of infection due to human parvovirus B19 may exacerbate anaemia among children who had already have anaemia due to other causes.

That a majority of anaemic pre-school children tested negative for human parvovirus B19 infection points to other aetiologies as the cause of anaemia in these children. In the tropics, anaemia may be due to iron deficiency resulting from insufficient dietary intake in children. In Nigeria, the majority of hospital admissions among children younger than the age of five years have been attributed to malaria.^14^ Malaria is thought to be the primary cause of severe anaemia in at least 50% of people living in malaria-endemic areas.^5^ It has also been shown that parvovirus B19 infection can play a critical role in the aetiology of severe anaemia in areas that are highly endemic for malaria.^15^

Our results also have implications for children living in the sickle cell belt of the tropics. Nigeria has a carrier rate for the sickle cell gene of 20%–30%, and sickle cell disease affects 2%–3% of the Nigerian population of more than 160 million people.^6^ Sickle cell anaemia may well become a life-threatening complication in patients infected with parvovirus B19. The presence of parvovirus B19 infection may exacerbate anaemia in children that have sickle cell disease.^16^

Among the 115 non-anaemic controls in this study, 18 (15.7%) tested positive for human parvovirus B19 infection. It is possible that these children had been exposed to parvovirus B19 recently and that they had recovered from infection-related anaemia prior to enrolment in the study. It could also be that the children who tested positive were truly anaemic but their haematocrit values had not risen to the cut-off value used in this study.

### Limitations

There are some limitations to our study, which should be noted. We were unable to perform PCR, qPCR or other genotypic testing for parvovirus B19 due to limited resources. Additionally, the dynamics of recovery from anaemia as a result of rapid erythropoiesis in healthy infected children and the impact of other anaemia-causing factors may have influenced results obtained in this study.

### Recommendations

There is an urgent need to initiate specific public health interventions to prevent anaemia and its attendant consequences in children. Appropriate screening for parvovirus B19 with IgM antibody detection and nucleic acid detection would exclude this viral agent during differential diagnosis of anaemia common in this age group.

### Conclusions

The high prevalence rate found in our study shows that human parvovirus B19 infection is common in this population of pre-school children.
